# A233 CHARACTERIZATION OF THE DUODENAL MICROBIOME IN PEDIATRIC CELIAC AND INFLAMMATORY BOWEL DISEASE PATIENTS

**DOI:** 10.1093/jcag/gwab049.232

**Published:** 2022-02-21

**Authors:** J Manoogian, J Butcher, J Li, D R Mack, A Stintzi

**Affiliations:** 1 University of Ottawa Faculty of Medicine, Ottawa, ON, Canada; 2 Children’s Hospital of Eastern Ontario, Ottawa, ON, Canada

## Abstract

**Background:**

Both Celiac disease (CE) and inflammatory bowel disease (IBD) are lifelong gastrointestinal tract (GIT) disorders. CE is an autoimmune disorder triggered by gluten consumption and can result in small intestine villus atrophy, crypt hyperplasia and epithelial permeability, while IBD is characterized by chronic, reoccurring inflammation and ulcers in the GIT. There is increasing evidence linking GIT microbes to both CE and IBD pathogenesis. Studies have also shown that CE patients are at increased risk of developing IBD, suggesting a possible link between these diseases. The duodenum can be affected in both CE and IBD, but it is understudied as compared to other GIT regions.

**Aims:**

We thus aimed to characterize the duodenal microbiome of pediatric CE, IBD and control patients (n=76, 48 and 57 respectively) and hypothesized that the composition of microbes would vary between each diagnostic group.

**Methods:**

We used mucosal luminal interface aspirates collected during upper endoscopy at the Children’s Hospital of Eastern Ontario. Metagenomic DNA was extracted from these samples and bacterial taxa was characterized by sequencing the V6 hypervariable region of the 16S rRNA gene.

**Results:**

Control, CE, and IBD duodenal microbiotas showed no apparent differences in either α-diversity or β-diversity. However, we identified several significant differences between the relative abundances of specific taxa in these three patient groups. In particular, *Atopobium parvulum* was found to be enriched in Crohn’s disease samples when compared to non-IBD controls. There was also a trend for higher *Bacteroides*, *Lactobacillus*, *Parabacteroides* and *Staphylococcus* in CE patient MLI aspirates. These results are similar to what has been previously reported in CE patients. Finally, trends found in the duodenal MLI aspirates are more consistent with results previously found in the salivary microbiome in CD patients as opposed to studies of the large intestine.

**Conclusions:**

This work provides unique insight into the microbial composition at the duodenum; a GIT region that has not been fully characterized in CE or IBD.

**Funding Agencies:**

CIHRGenome Canada

